# *Craticula scientiacivica* (Stauroneidaceae), sp. nov., a new diatom species found during a citizen science project

**DOI:** 10.3897/phytokeys.275.192720

**Published:** 2026-05-28

**Authors:** Jennifer Moyón, María Borrego-Ramos, Ángela Taboada, Adriana Olenici, Saúl Blanco

**Affiliations:** 1 Laboratorio de Diatomología, Instituto de Medio Ambiente y Cambio Global (IMACG), Universidad de León, La Serna 58, 24007, León, Spain Faculty of Environmental Sciences and Engineering, Babes-Bolyai University Cluj-Napoca Romania; 2 Departamento de Biodiversidad y Gestión Ambiental, Facultad de Ciencias Biológicas y Ambientales, Universidad de León, León 24071, Spain Instituto de Medio Ambiente y Cambio Global (IMACG), Universidad de León León Spain; 3 Faculty of Environmental Sciences and Engineering, Babes-Bolyai University, Fantanele Street, No. 30, 400294, Cluj-Napoca, Romania Facultad de Ciencias Biológicas y Ambientales, Universidad de León León Spain

**Keywords:** Citizen science, new diatom species, panduriform valves, Stauroneidaceae, teratological forms

## Abstract

*Craticula scientiacivica* Moyón & S. Blanco, **sp. nov**., is a new diatom species discovered during the CiDIA-micro citizen science project at the University of León, Spain, which evaluates the environmental impact of biodegradable packaging on periphytic diatom communities. This initiative involved secondary school students and teachers in sampling experimental aquaria inoculated with natural benthic algae from Laguna Sentiz (León, Spain), revealing the novel taxon in a control treatment under alkaline conditions (pH 9, low nutrients). Valves are linear-lanceolate, panduriform, with concave margins and protracted rostrate apices, measuring 32.0–35.7 μm long and 3.9–6.3 μm wide. Striae are uniseriate, 18–19 in 10 μm, and weakly radiate, with circular central areolae (37–46 in 10 μm) transitioning to elliptical forms apically. The raphe is filiform, with expanded, unilaterally deflected proximal endings and terminal hooks extending onto the mantle. Teratological forms were found in 26% of individuals, featuring deformed outlines, misaligned raphe, and aberrant copulae, possibly linked to culture conditions despite the absence of chemical stressors. Morphologically, *C. scientiacivica* aligns with *Craticula* (Stauroneidaceae) via its shallow mantle, apically elongated external foramina, and deflected central raphe endings, but its unique panduriform shape and dimensions distinguish it from congeners. Molecular data are pending, but its occurrence in a single experimental tank suggests aerial propagule dispersal. This discovery underscores the role of citizen science in uncovering hidden biodiversity, bridging education and taxonomy in diatom research.

## Introduction

The diatom genus *Craticula* Grunow was erected to accommodate species formerly in *Navicula* sect. *Orthostichae*, and late 20^th^-century morphological reassessments and ultrastructural studies reinstated this section as a separate genus. Key morphological traits include a raised axial area, often with a conopeum; dense striae; and, in many species, the formation of heavily silicified “craticular valves” under stress. Within *Craticula*, species differ in valve shape and dimensions, stria and areola patterns, and ultrastructural details (Mann and Stickle 1991). The frustules are isovalvar, isopolar, symmetric, and biraphid with various outlines. The raphe structure is characterized by a thick raphe-sternum, straight to deflected central raphe fissures, and hook-like terminal endings. Striae are uniseriate and composed of round to elliptical areolae occluded internally by hymens. The valve outline is variable, from lanceolate to rhombic, with protracted, subrostrate, or subcapitate apices in many species. A linear, narrow axial area with a typically slightly dilated or linear-lanceolate central area is usually visible in light microscopy (LM). Striae tend to be parallel throughout the valve, regular, and sometimes terminating before the apices (Beauger et al. 2019; Blanco et al. 2019; Solak et al. 2020). Certain species bear conspicuous longitudinal ribs or unusual siliceous coverings on the valve surface observable in scanning electron microscopy (SEM).

Molecular studies place *Craticula* in the Stauroneidaceae clade along with *Stauroneis* Ehrenberg, *Prestauroneis* K.Bruder & Medlin, *Fistulifera* Lange-Bertalot, *Parlibellus* E.J.Cox, and allies (Kulikovskiy et al. 2019). Genera such as *Mayamaea* Lange-Bertalot and *Sellaphora* Mereschkowsky, which were once considered morphologically similar, actually fall on a separate phylogenetic branch (Zgrundo et al. 2013). However, species currently assigned to *Craticula* fall into at least two well-supported molecular clades, and there is at present no strong support for monophyly of the genus (Kulikovskiy et al. 2019). By transferring the generitype of *Lacunicula* (*L. sardiniensis*) Lange-Bertalot, Cavacini, Tagliaventi & Alfinito to *Craticula*, Morales and Le (2005) synonymized both genera, with priority to *Craticula*. Six new species have been described in the last decade, from Antarctica (*C. australis*, *C. obaesa*, and *C. petradeblockiana* Van der Vijver, Kopalová & Zindarova), Senegal (*C. widouensis* Beauger, C.E.Wetzel & Ector), Turkey (*C. anatoliana* Solak, M.Rybak & Peszek), and Spain (*C. gadorensis* Blanco), expanding the genus to 60 species, seven varieties, and two forms.

Ecologically, most *Craticula* species are stress-tolerant, favoring saline, brackish, polluted, or mineral-rich habitats, with a broad but patchy distribution from Europe to the Antarctic. Many species have been recorded from temporary (ephemeral) ponds and hydrothermal/thermal springs, showing affinity for transient or thermally influenced habitats in some taxa (Morales et al. 2014; Blanco et al. 2019).

During a citizen science project involving the participation of students and teachers from secondary schools together with senior scientists at the Institute for the Environment and Global Change (IMACG, León, Spain), an unknown diatom was found occurring in one of the experimental aquaria. This paper describes this population with detailed micrographs and an exhaustive comparison with similar taxa, proposing it as a new species within the genus *Craticula*.

## Materials and methods

The CiDIA-micro project, framed within the 2024 Microprojects of the European University Alliance EURECA-PRO at the University of León (Spain), assesses the aquatic environmental impact of biodegradable and compostable packaging materials (certified under standards UNE-EN 13432:2001 and TÜV Austria OK compost HOME) through a citizen science approach (Borrego-Ramos et al. 2025). This initiative engages local educational communities (111 students and seven teachers from six high schools) in the collection and analysis of water samples to monitor alterations in diatom communities. The experiment consists of the culture of benthic algae in 18 L tanks for 20 weeks to assess the effect of different bioplastics on the growth of periphytic diatoms, measuring different response variables (abundance, diversity, and occurrence of teratological forms). The material analyzed in this study comes from one of the experimental units set at the secondary school IES Claudio Sánchez Albornoz (León).

Water parameters, including pH and chemical variables—ammonium, nitrites, nitrates, carbonate hardness, and phosphates—were determined *in situ* with a VISOCOLOR ECO reagent kit (Altmann Analytik GmbH & Co. KG). Periphytic algae growing on artificial substrata (7 cm^2^ tiles) were sampled using a toothbrush and preserved with 4% (v/v) formaldehyde. In the laboratory, a suspension of cleaned diatom frustules was prepared by oxidizing the organic material with 30% (v/v) hydrogen peroxide, heating the solution to 70–90 °C to enhance the reaction. Hydrochloric acid (3 M) was subsequently added to eliminate calcium carbonate residues. LM slides were mounted in Naphrax®, a synthetic resin with a high refractive index. Identification and quantification of diatom taxa were performed at 1000× magnification with a DIC-equipped Olympus BX60 light microscope coupled to an OPTIKA camera. For SEM analysis, a drop of the cleaned sample was placed on a conductive metal stub, air-dried, coated with a 10 nm gold layer using a QUORUM Q150T ES metallizer, and examined at the Electron Microscopy Unit of the University of Jaén (Spain) using a MERLIN (Carl Zeiss) microscope operating at 20 kV. Image processing was conducted with GIMP software.

## Results

### Craticula scientiacivica

Taxon classification

Plantae

DipteraSarcophagidae

Moyón & S. Blanco sp. nov.

4A31464A-A182-5F9E-902A-5E2446B29A4C

Figs 1, 2, 3

#### Holotype.

Slide LEB DIATOMEA 31-1, deposited in the Herbarium Jaime Andrés Rodríguez. ***Isotype***. slide IMACG_01 (IMACG). Registration: http://phycobank.org/107308.

**Figure 1.**
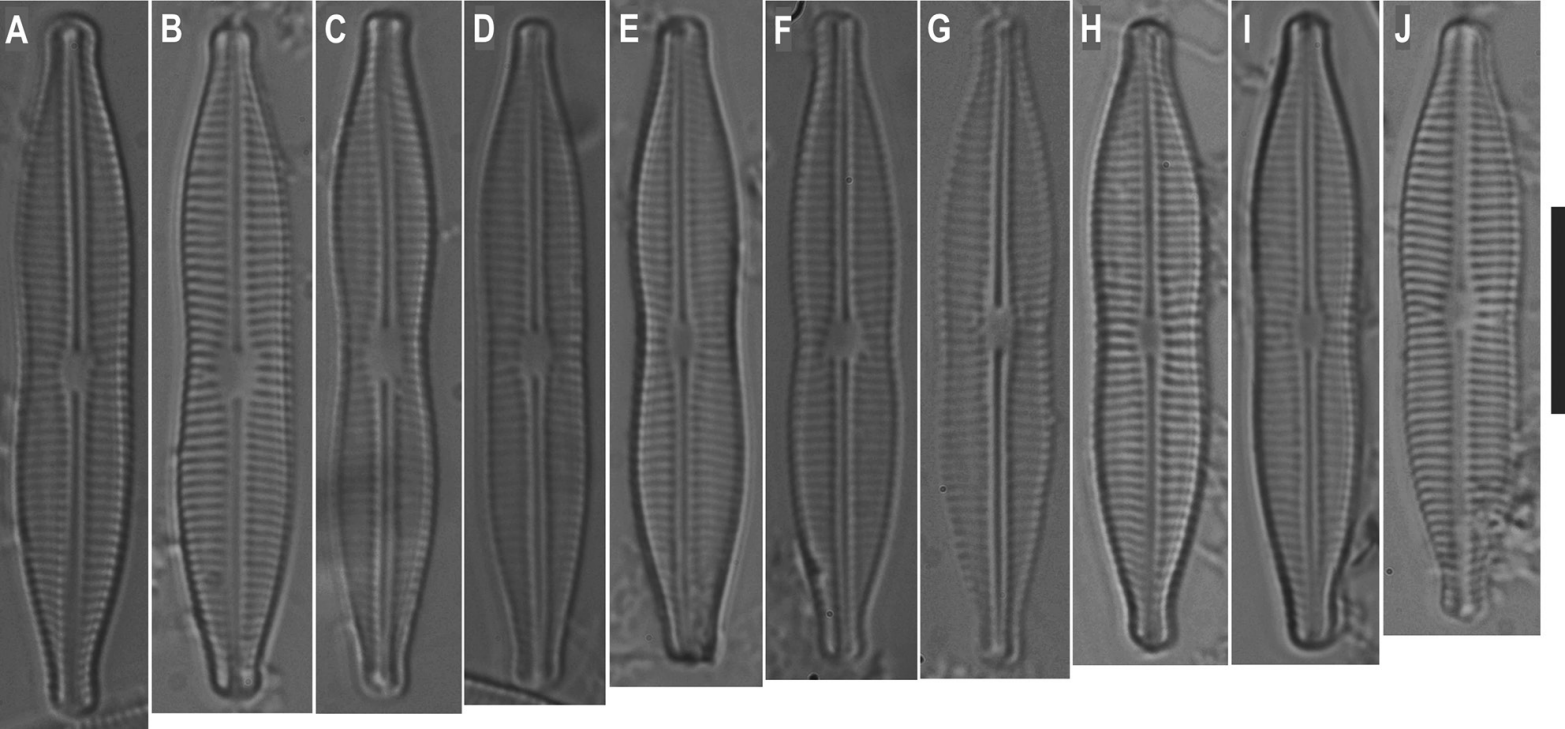
**A–J**. LM of *Craticula scientiacivica* sp. nov. from the holotype slide. Scale bar: 10 μm.

**Figure 2.**
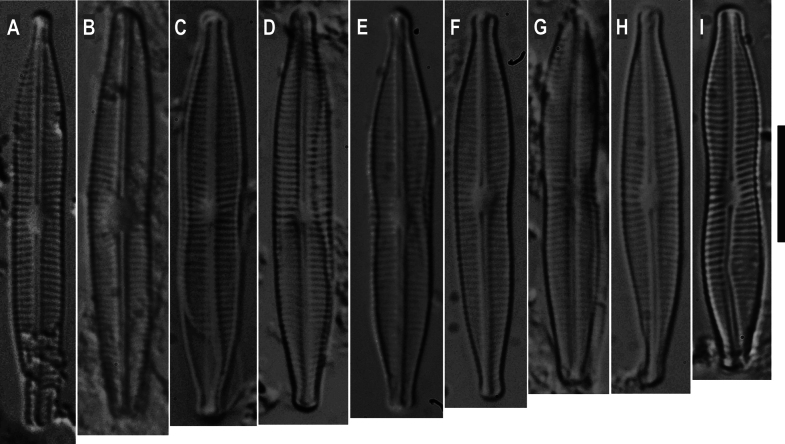
**A–I**. LM showing teratological forms of *Craticula scientiacivica* sp. nov. from the holotype slide. Scale bar: 10 μm.

**Figure 3.**
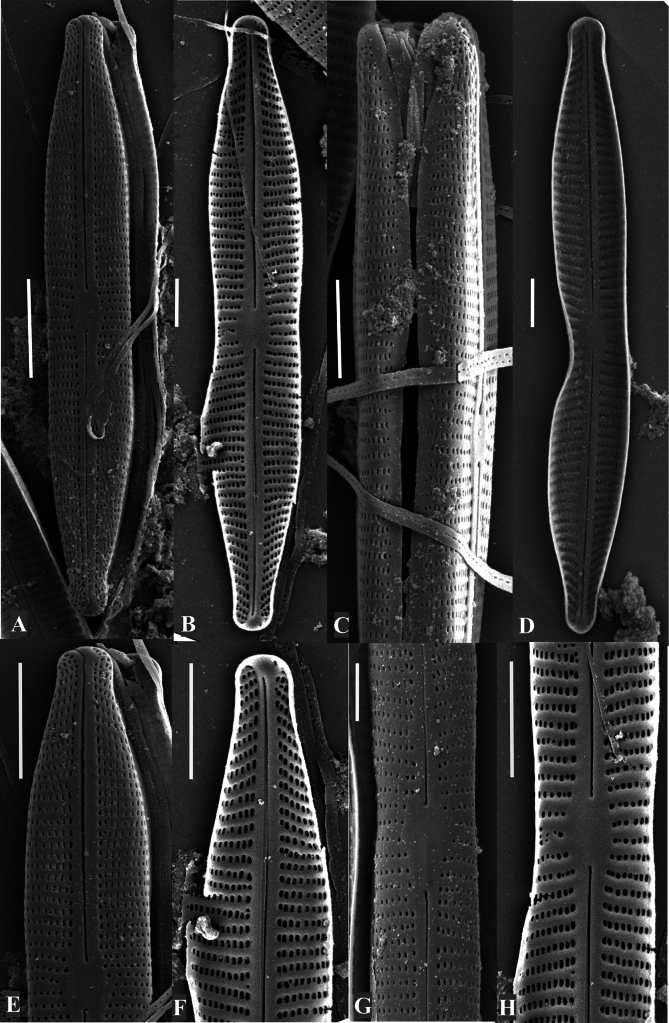
SEM of *Craticula scientiacivica* sp. nov. **A**. External view of the entire valve; **B**. Internal view of the entire valve; **C**. Girdle view; **D**. Teratological valve; **E**. External view of rostrate ends; distal ends hooked and continuing onto the valve mantle; **F**. Internal view of rostrate ends, distal end with helictoglossa developed; **G, H**. Circular central area, proximal raphe endings slightly deflected toward one side of the valve, and uniseriate striae composed of circular areolae at the valve center. Scale bars: 2 µm (**A–F, H**); 1 µm (**G**).

#### Type locality.

Spain • León Province, experimental freshwater aquarium at IES Claudio Sánchez Albornoz Institute, inoculated with epiphytic algal communities collected from Sentiz Lake, Valdepolo, 42°33'36"N, 5°12'36"W, 920 m a.s.l., 24 Jan. 2025, DiatomLab members leg., epiphytic material obtained by scraping roots of *Myriophyllum alterniflorum* DC in Lam.

#### Description.

**LM** (Fig. 1). Valves linear-lanceolate with concave-contracted margins at the mid-valve and protracted, rostrate ends (Fig. 1A–J). Valve dimensions (n = 25): Length 32.0–35.7 µm; width 3.9–6.3 µm. Striae visible, 18–19 in 10 µm, weakly radiate.

**SEM** (Fig. 3). Axial area narrow and linear, central area circular (Fig. 3G, H). Raphe straight, filiform. Externally, proximal raphe endings expanded into central pores and slightly deflected to the same side (Fig. 3A, G); internally, almost straight (Fig. 3B, H). Distal raphe endings externally hooked, reaching the valve mantle at the apex (Fig. 3E). Internally, helictoglossae developed and distal ends slightly bent on the same side (Fig. 3F). Striae slightly curved around the central area and convergent at the ends; uniseriate (Fig. 3A, B). Internally, areolae circular at the valve center (37–46 in 10 µm; Fig. 3H); becoming elongated and elliptical toward the ends (18 in 10 µm; Fig. 3F); but linear in external view (21 in 10 µm; Fig. 3E). In girdle view, areolae continuing onto the mantle (40 in 10 µm; Fig. 3C). “Craticula” or “heribaudii” stages not seen. Teratological forms were frequently observed (26% of the population, *n* = 96 out of total valves), including abnormal valve outlines characterized by deflected ends, absence of typical median constriction on one side, deformed copulae, and raphe modifications such as misalignment (Figs 2A–I, 3D).

#### Etymology.

The specific epithet scientiacivica refers to its discovery during the development of a citizen science project involving secondary schools.

#### Ecology and distribution.

*Craticula scientiacivica* was found under controlled experimental conditions in microcosms inoculated with material obtained from root scrapings of *Myriophyllum alterniflorum*. Although the species occurred across different microcosms, the highest population abundance was recorded in the control treatment. Environmental conditions were characterized by a pH of 9, a hardness of 1.3 °dH, an ammonium of 0.1 mg L^-1^, and a phosphate of 3 mg L^-1^; nitrite and nitrate concentrations remained below the detection limit (< LOD). During light microscopy (LM) observations, *C. scientiacivica* was found to be associated with *Achnanthidium minutissimum* (Kützing) Czarnecki, *Craticula buderi* (Hustedt) Lange-Bertalot, *Encyonopsis microcephala* (Grunow) Krammer, *Navicula radiosa* Kützing, *Nitzschia amphibia* Grunow, *N. paleacea* (Grunow) Grunow, *N. tenuirostris* Manguin, *Gomphonema acuminatum* Ehrenberg, *G. capitatum* Ehrenberg, *G. subclavatum* (Grunow) Grunow, *Punctastriata lancetulla* (Schumann) P.B.Hamilton & Siver, *Stephanocyclus meneghinianus* (Kützing) Kulikovskiy, Genkal & Kociolek, *Ulnaria ulna* (Nitzsch) Compère, and *U. biceps* (Kützing).

## Discussion

The new species documented here can be assigned to the genus *Craticula* based on the presence of distinctive features such as a shallow mantle (Morales et al. 2014); apically elongated external areola foramina that become roundish at the valve center together with elliptical internal areolae, and not lineolae as in *Navicula* (Mann and Stickle 1991); and external central raphe endings turned slightly toward the primary side of the valve (Fig. 3A). *Craticula scientiacivica* lacks longitudinal ribs and a thick raphe-sternum, but these characteristics are also absent in many other *Craticula* taxa, such as *C. accomoda* (Hustedt) D.G.Mann, *C. subminuscula* (Manguin) C.E.Wetzel & Ector, and *C. molestiformis* (Hustedt) Mayama, and thus seem not to be apomorphic. The typical “Craticula” and “Heribaudii” forms that occur under stress conditions during the formation of the resting spore were also not detected in the material.

The combination of valve shape and morphometric data in *C. scientiacivica* seems to be unique among *Craticula* taxa. To our knowledge, this is the only species within the genus with a consistent panduriform shape in frustule outline. The morphologically similar *Navicula akebergii* Kützing shows a distinctive stigma in the central area that prevents its association with the genus *Craticula*. *Navicula adamsii* Cholnoky from Lake Tanganyika also has a slightly constricted outline in the midvalve, but the apices are much less protracted than in *C. scientiacivica*. Other *Craticula* species are barely comparable to the new species described here based on valve dimensions or shape (Suppl. material 1).

The type population of *C. scientiacivica* was found to be prone to develop teratological forms, which occurred in 26% of the individuals observed. Several diatom genera are frequently noted for their tendency to produce aberrant valves, such as *Amphora*, *Encyonema*, and *Pseudo-nitzschia*, especially in polluted environments or laboratory cultures. In this sense, the 20-week duration of this study could have played a significant role, as an increase in teratological forms has been linked to prolonged cultivation time (Mohamad et al. 2022). In the case of *C. scientiacivica*, deformed cells dominated in control treatments, ruling out the leaching of chemicals from bioplastics as a factor. However, unfavorable culture or nutritional conditions have been reported to induce teratologies in *Navicula* species in laboratory/aging cultures (Falasco et al. 2021; Petrova et al. 2025). Although all treatments and replications were inoculated with the same aliquot coming from a natural environment, *C. scientiacivica* was recorded in nine out of the 17 microcosms. Furthermore, the species reached its maximum development in a single control tank, where 96 individuals were observed. This leads to the hypothesis that this species may have arrived in the form of aerial propagules and grew accidentally in one of the aquaria curated by the students.

*Craticula scientiacivica* is not the first diatom species discovery reported from artificial tanks. For instance, Monnier et al. (2003) described *Nupela exotica* in a tropical freshwater aquarium near Orleans (France), and *Eunotia pottieziana* was found living in a tropical aquarium in the author’s residence (van de Vijver 2023). It is also not the first species discovered as a result of a citizen science project, as Bahls (2014) documents 12 new taxa collected by citizen volunteers from remote locations in the western United States. Citizen science is now a major source of biodiversity data, particularly species occurrence records at large spatial scales, which can reveal poorly known taxa and unexpected distributions (Encarnação et al. 2021; Feldman et al. 2021). In particular, school-based citizen science projects usually involve students in data collection in the fields of ecology and biodiversity monitoring but rarely report direct new species discoveries because of the lack of expert taxonomists and infrastructure for feedback and validation. An example could be the School Malaise Trap Program (Leerhøi et al. 2024), which generated the first barcode records for hundreds of species, illustrating how school programs can feed large biodiversity initiatives. Similarly, in the “Hidden World of Bacteria” project (Pitt et al. 2020), school classes worked with researchers to isolate and describe new bacterial strains from inland waters. Students actively participated in sampling and handling and even contributed to the publication of four peer-reviewed articles describing new genera and species. The finding of *C. scientiacivica* thus illustrates how citizen science has demonstrable power to uncover new species, including diatoms, when school experiments are integrated with professional taxonomic work.

## Supplementary Material

XML Treatment for Craticula scientiacivica

## Additional information

### Artificial Intelligence (AI) use

The authors accept full responsibility for the content of the manuscript, including the disclosure of any use of AI.

No AI tools were used in the preparation of this manuscript.

### Author ORCIDs

J. Moyón https://orcid.org/0000-0002-9164-6696

M. Borrego-Ramos https://orcid.org/0000-0002-0895-1623

Á. Taboada https://orcid.org/0000-0002-3494-4806

A. Olenici https://orcid.org/0000-0001-5554-6236

S. Blanco https://orcid.org/0000-0002-9015-2512

### Data availability

All of the data that support the findings of this study are available in the main text or Supplementary Information.

## Supplementary materials

Supplementary material 1 Morphological comparison Data type xlsx Explanation note **table S1**. Morphological comparison between *Craticula scientiacivica* sp. nov. and morphologically similar taxa. https://binary.pensoft.net/file/1660435This dataset is made available under the Open Database License (http://opendatacommons.org/licenses/odbl/1.0/). The Open Database License (ODbL) is a license agreement intended to allow users to freely share, modify, and use this Dataset while maintaining this same freedom for others, provided that the original source and author(s) are credited. Jennifer Moyón, María Borrego-Ramos, Ángela Taboada, Adriana Olenici, Saúl Blanco
